# Early Access to Medicines: Use of Multicriteria Decision Analysis (MCDA) as a Decision Tool in Catalonia (Spain)

**DOI:** 10.3390/jcm11051353

**Published:** 2022-03-01

**Authors:** Montse Gasol, Noelia Paco, Laura Guarga, Josep Àngel Bosch, Caridad Pontes, Mercè Obach

**Affiliations:** 1 Department of Pharmacology, Therapeutics and Toxicology, Universitat Autònoma de Barcelona, 08193 Bellaterra, Spain; montserrat.gasol@autonoma.cat; 2 Catalan Health Service (CatSalut), 08007 Barcelona, Spain; npaco@catsalut.cat (N.P.); laura.guarga@catsalut.cat (L.G.); mobach@catsalut.cat (M.O.); 3 Digitalization for the Sustainability of the Healthcare System (DS3), Bellvitge Biomedical Research Institute (IDIBELL), 08006 Barcelona, Spain; 4 Department of Medicine, Universitat Autònoma de Barcelona, 08193 Bellaterra, Spain; jaboschg@gmail.com

**Keywords:** early access, multicriteria decision analysis, MCDA, drug policy, assessment, pharmaceuticals

## Abstract

Early access to medicines allows the prescription of a medicine before it is available in the public formulary to patients with severe or rare diseases with high unmet needs who have no authorised therapeutic alternatives available. In this context, consistent decision making is difficult, and a systematic assessment procedure could be useful to tackle complex situations and guarantee the equity of medicines’ access. A multidisciplinary panel (MP) conducted four workshops to develop an early access framework based on a reflective multiple criteria decision analysis (MCDA). A set of 12 criteria was agreed: eight quantitative (severity of disease, urgency, efficacy, safety, internal and external validity, therapeutic benefit and plausibility) and four qualitative (therapeutic alternative, existence of precedents, management impact and costs). Quantitative criteria were weighted using a five-point scale. The relative importance of quantitative criteria had mean weights from 4.7 to 3.6, showing its relevance in the decisions. The framework was tested using two case studies, and reliability was assessed by re-test. The re-test revealed no statistical differences, indicating the consistency and replicability of the framework developed. MCDA may help to structure discussions for heterogeneous treatment requests, providing predictability and robustness in decision making involving sensitive and complex situations.

## 1. Introduction

Medicines in European countries require a marketing authorisation granted to confirm quality, efficacy and safety before they can be placed on the market [1,2]. Additionally, other milestones have to be reached before the medicine is commercially available to patients, including health technology assessment (HTA) and decisions on pricing and reimbursement (P&R) [2,3,4]. HTA measures the added value of a new health technology compared to existing ones to formulate health policies and support decisions about coverage at a national level. For this, HTA assesses the best evidence on relative efficacy/effectiveness, safety, relevance to the quality of life and economic aspects [2,4,5]. Thus, post-authorisation procedures aim to guarantee the provision of treatments with added value and benefit to the maximum number of patients while ensuring public health care sustainability [5].

In order to optimise patient access to disruptive innovation, the European Medicines Agency (EMA) has implemented pathways to accelerate access to market to medicines addressing unmet needs, such as the priority medicines scheme (PRIME). Additionally, approvals under special circumstances (conditional or exceptional) rely on less comprehensive data at the time of decision than normally required, with missing information being collected at later stages [6]. Through lowering the standard of evidence required for authorisation, new challenges arise to HTA bodies and payers, who now lack robust data and face substantial uncertainty to decide in regard to efficacy, safety and cost impact.

The unprecedented speed of new medicine’s approval in recent years parallels increasing prices, uncertainties in clinical benefit and comparative effectiveness and cost-effectiveness, and progressively constrained budgets. Thus, policy and health decision-makers have to tackle to balance multiple objectives of health care systems: access to innovation, fairness and equity while ensuring efficiency and sustainability [7]. This complex scenario has prolonged the duration of the final access phase, leading to what is referred to as market access barriers [7]. The gap between the time the medicine is approved by a regulatory agency and the moment is commercially available for prescription can be notably long, and the scientific literature indicates significant timely differences between European countries, especially in orphan drugs and oncology treatments [3,4,8].

Delay in access becomes an ethical dilemma in clinical areas for which therapeutic needs are huge [9,10] and for people with serious, debilitating or life-threatening diseases without available treatment options due to comorbidities, exhaustion of all treatment options, adverse events, among others [10,11]. Lack of patient early access to promising innovation may represent huge individual opportunity costs when available information suggests that accessing a treatment earlier in the progression of a patient’s pathology may avoid health deterioration, which will otherwise occur due to long waiting times until official market authorisation. To fulfil special needs and to overcome access barriers to new promising medicines in severe conditions with high unmet medical needs, early access to medicines allows their provision before completing all the regulatory and/or access decisions [3,12]. To this end, most countries have implemented some mechanisms as a gateway to the prescription of medicines when they are not available through standard procedures [12].

In Spain, the Royal Decree (RD) 1015/2009 regulates the availability of medicines in special situations (MSS) [13]. The RD establishes the following: (1) requirements for compassionate use of medicinal products in clinical research for patients who are not part of a clinical trial; (2) conditions for off-label use of medicines; and (3) access through importation to unauthorised medicines in Spain provided that they are lawfully authorised or already marketed in other countries. In all of the previous circumstances, the RD states the exceptionality of these medicines usage by limiting it to patients with no therapeutic alternatives authorised. Although not foreseen in the RD, managing new indications for which public coverage decisions have not yet been made is also a challenging situation [14]. The need to incorporate the “pre-reimbursed” (i.e., not yet covered) indications in the RD has been previously highlighted [15]. In order to meet the conditions set out in the RD for MSS use, hospitals perform an evaluation of each case through the Pharmacy and Therapeutic Committees (PTC) and an individual authorisation is issued [16].

While the clinical benefits that early access can bring to those patients in need are recognised, the use of MSS and pre-reimbursed indication may generate strains in the system, as their financial coverage is not clearly established in the Spanish legislation. In addition, the individual nature of the decisions and the atomised and uncoordinated assessments carried out in different healthcare centres by the PTC may be a source of inequity since the treatment indication decision may differ between them. In addition, the treatment cost is often high and non-negotiable at this stage of the procedure, which increases the financial burden to hospitals in a way unpredictable to foresee. The acceptance of the marketing authorisation holder price at this point could perfectly interfere with the P&R procedure and decision afterwards. In rare diseases, consecutive decisions may lead to a substantial proportion of the target population being treated at high foreign prices so that there is no incentive to request or accept lower local pricing to the company. Additionally, the possibility that the medicines/indication is finally financed but with some restrictions (sub-populations or sub-indications) [4] or finally not reimbursed in Spain can generate access inequity between patients who have been treated in an early-access stage versus those that are treated after P&R decision is made. Moreover, clinicians’ relative unfamiliarity with the regulations can lead to inappropriate access requests, and therefore, health managers have to deal with clinicians’ and patients’ expectations. Situations as previously described are a source of confusion and discomfort among physicians, patients and payers.

Some European countries have developed programmes for drug access before a P&R is decided, such as the French Temporary authorisation for use (ATU) or the early access to medicines (EAMS) in the UK. The ATU programme has been running in France since 1994 and allows access before the completion of P&R negotiations. Access to pre-authorised medications in France may be granted through the nominative ATU (nATU: for an individual patient) or cohort ATU (cATU: for a group of patients). However, as a result of the expanded use of cATUs over recent years and the growth of a dedicated budget in 2020, the French Ministry of Health proposed several reforms to the ATU System showing its challenges and need to sort and delimit. However, to our knowledge, a specific methodology for the assessment of these situations has not yet been reported.

In Catalonia, an informal assessment process of individual requests for MSS was customary until 2018. Lack of standardised procedure could lead to inconsistency, variability, lack of predictability and even transparency of decisions and thereby question their legitimacy. To redress these challenges and equity risks, the Catalan Health Service (CatSalut) created the Advisory Committee of Medicines in Special Situations (CAMSE, for its acronym in Catalan) in 2018 [17]. CAMSE is a multidisciplinary board whose composition is determined by local regulations and includes different professional backgrounds and patient representatives to provide a holistic approach to MSS decision-making. The main objectives of CAMSE are to evaluate and draw up coordinated proposals or recommendations of use among hospital committees, periodically monitor the utilisation and healthcare outcomes of MSS, and advise on individual requests (i.e., case-by-case) for the use of non-authorised medicines and pre-reimbursed medicines/indications (from now on NAM-PR) in Catalonia.

To accomplish the last purpose, it was proposed that a structured approach involving multiple criteria may improve decision-making procedures [18]. Multicriteria decision analysis (MCDA) enables a holistic assessment by a systematic, transparent and explicit consideration of multiple aspects beyond the traditional criteria used by HTA [18,19,20]. This methodology has been used to support different types of decisions and at different levels and contexts: to set priorities by HTA among alternatives at the national level or by PTC in the hospital setting, to support decision making about pricing and reimbursement or to assist clinical decisions in specific areas such as oncology or orphan drugs [21,22,23,24,25,26,27]. The Evidence and Value: impact on decision making (EVIDEM) is a reflective MCDA approach that provides an adaptable framework that has been used in a variety of healthcare settings to support the deliberative process. The framework provides a structured consideration of criteria and a standardised process promoting the reflection of the stakeholders while sharing their diverse perspectives [20,21,28].

Thus, the objective of the present study was to develop a reflective MCDA for the assessment of individual early access medicines request to support decision making in CatSalut.

## 2. Materials and Methods

The reflective MCDA (EVIDEM) was selected as the reference multicriteria framework. Four sessions were planned for the qualitative study using a stepwise approach: (1) selection and structuration of quantitative (core model) and qualitative (contextual tool) criteria; (2) weighting of the quantitative criteria and testing the resulting framework through assessment of one case study and refinement if necessary; (3) final validation through assessment of a second case study; and (4) re-testing the framework after 6 months of implementation in real setting by the CAMSE.

### 2.1. Panel Design and Conduct of the Sessions

Eighteen panellists were invited to participate. Because the aim was to provide systematic methods to the activity that the CAMSE was already undertaking, the panel included all of its members (*n* = 16): 3 policymakers, 5 clinicians (1 of them with bioethical background), 7 current members of PTC from 7 different hospitals (4 clinical pharmacologists and 3 hospital pharmacists) and 1 patient representative. Additionally, one economist and an additional policymaker from CatSalut were invited to collect perspectives on the final steps of decision making by managerial staff, based on the assessments of CAMSE. The panellists were classified as policymakers, evaluators, clinicians or patient representative.

The secretary of the Advisory Committee chaired the sessions, and two experienced investigators trained the participants on reflective MCDA methodology.

### 2.2. Selection and Structuring Criteria

Before the development of the sessions, some criteria were preselected based on the decision problem and according to previously published studies in other settings [24,27,29]. The selected criteria for the framework were based on MCDA Emerging Good Practices Task Force and the reflective MCDA approach. Both consider requirements of completeness, non-redundancy, non-overlap, and preferential independence of the criteria [28,30].

Based on previous MSS assessment, taking into account the recommendations of the legislation for MSS use, and considering the approach of case-by-case requests, the investigators identified, included and adapted some relevant criteria in the pilot framework for evaluating MSS.

The panellists gave their insights about the proposed criteria and subcriteria, agreed upon their definitions, and voted for the inclusion, inclusion with modifications or exclusion of the criteria in the framework. The consideration of the qualitative or quantitative type of criteria was also discussed.

Criteria were selected by consensus so that more than 50 per cent of the panellists had to vote “yes” to include a criterion in the framework or “no” to exclude it. When other combinations of answers were obtained, criteria were adapted. Once agreed, the participants elicited the scores scales.

### 2.3. Weighting of Criteria of Early Access Framework

The weighting of the quantitative criteria of the framework was done using a non-hierarchical 5-point scale. All panellists gave a relative weight per criteria, where 1 meant lowest relative importance and 5 highest relative importance [28,30]. Criteria weights were normalised to sum up to 1 for each participant: for the 5-point rating scale method, each weight was divided by the sum of weights across all criteria; for the point allocation method, criteria ratings were multiplied by domain weight and rescaled to range from 0 to 1 [27].

If relevant changes were implemented in the pilot version after testing, the framework weighting exercise was done again.

### 2.4. Case Studies

Two different types of medicines and individual requests of MSS were chosen to test the suitability of the proposed MCDA framework.

The first one was a request for burosumab, an orphan drug that was authorised but not commercialised in Spain for the treatment of a patient with X-linked hypophosphatemia (rare disease). The drug was requested for use within the authorised indication, but before the Spanish Ministry of Health decision on P&R, therefore the medicine had to be acquired abroad and imported to Spain. The second one was a request for dupilumab for a paediatric patient with atopic dermatitis. Dupilumab was at that time reimbursed only in adults, with a restricted reimbursement as compared to the authorised label and a performance-based risk-sharing scheme [31]. The paediatric indication had been recently authorised and still undergoing the P&R process (pre-reimbursement new indication) in Spain. The available evidence was summarised for both drugs in the evidence matrix, following the EVIDEM methodology [28].

During the second and third session, the participants rated the case studies individually in the evidence matrix by assigning a score and including comments to each criterion. The quantitative criteria were rated using a categorical (e.g., from 0 to 5) scale, while the qualitative ones were qualified as positive, negative or neutral impact.

The design and conduct of the study were aligned with good practice guidelines on the use of MCDA in health care decisions [18,30].

### 2.5. Data Analysis

Data were collected individually via the Socrative application and analysed with Microsoft Excel software. The value contribution (*VC_x_*) of each quantitative criterion was calculated as the product of its normalised weight (*W_x_*, ∑*W_x_* = 1) and standardised score (*S_x_* = score/number of possible categories). The overall MCDA value estimate (*VE*) of each case study is calculated based on a linear additive model as a sum of all criteria value contributions (*VC_x_*) of all (*n*) criteria of the quantitative criteria:

$$VE=\overset{n}{\underset{x=1}{\sum }}VC_{x}=\overset{n}{\underset{x=1}{\sum }}\left(W_{x}\times S_{x}\right)$$

### 2.6. Re-Test of the Early Access Framework

To assess the reproducibility of weights, scores and value estimates, a re-test was conducted 6 months after the development of the early access framework. Individually, the members of the CAMSE repeated a reflective MCDA for a real request of an orphan drug (volanesorsen) that had been discussed 6 months earlier. Volanesorsen is an orphan medicine authorised for the treatment of familial chylomicronaemia syndrome (FCS) not commercialised in Spain at the request moment and with high treatment cost as an imported medicine. The Wilcoxon singed-rank test was used to detect significant differences between the two assessments.

## 3. Results

### 3.1. Criteria Selection for Early Access Framework Individual Requests

The panel was composed of different professional profiles: 39% were evaluators, 28% clinicians, 28% policymakers and 5% patient representatives.

The investigators proposed a pilot framework with a mixed approach that consisted of a core model of eight quantitative criteria and a contextual tool with three qualitative criteria. Four criteria were extracted and two adapted from the EVIDEM framework v.4 (10th Edition) [27], while five criteria were introduced considering the early access special context and adapted for a case-by-case evaluation (Table 1):

- Medical urgency (quantitative criterion): The rationale for including this criterion was to assess whether a window of opportunity in prognosis was relevant for early access in order to avoid irreversible adverse consequences of the disease. Thus, the speed progression of the disease and/or the related complications at the time of the request and the appropriate moment to treat the patient to obtain a therapeutic benefit should be assessed (e.g., immediate intervention is needed because of a fulminant progression of the disease, or alternatively, the treatment could wait because progression is slow or reversible).
- Therapeutic alternatives (quantitative criterion): the use of MSS should be limited to situations where no therapeutic alternatives or no suitable alternatives are available for the individual patient, including clinical trials.
- Plausibility (quantitative criterion): the criterion assesses the credibility or probability that the treatment could cause a beneficial effect in the particular patient due to its mechanism of action.
- Existence of precedent and coherence of decisions (qualitative criterion): This criterion was proposed as a qualitative aspect to reflect the need for consistency and equity considering previous decisions taken, as well as the representatively of the case in relation to past or future requests.
- Influence of the decision at a policy level or management impact (qualitative criterion): The criterion reflected the potential interference of the early use of the medicine in the negotiations between the Ministry of Health and the marketing authorisation holder since the majority of requests are of pre-reimbursed medicines/indication.

All the panellists discussed and validated the pilot framework.

As a result of the discussion during the first session, all criteria in the pilot framework were maintained with some definitions improved for better comprehension. Nevertheless, during the second session, some modifications of the pilot framework were further agreed upon after testing the framework with the evidence matrix of burosumab. The criterion “quality of evidence” was redefined as “internal validity”, and a criterion of “external validity” was included while excluding it as a subcriterion of “plausibility”. The panellists considered it relevant to include “budget impact” as a subcriterion of “medical costs” given the relevance on future economic consequences of early access of NAM-PR and as an indirect way of informing the size of the affected population that could be suitable for treatment under special circumstances. Finally, the “therapeutic alternative” criterion was transferred to the contextual tool.

Regarding the scoring of quantitative criterion, a categorical scale from 0 (worst, low or none) to 5 (best or high) was agreed, except for the “plausibility” criterion, which was a dichotomous scale (0—plausibility exists or 5—not plausible).

After the reflective discussion, the panellists agreed on a final framework that included eight quantitative criteria (core model) and four qualitative criteria (contextual tool). Table 2 shows the final criteria, subcriteria and scoring scales (see Table S1 for full definitions of the criteria).

### 3.2. Weighting of Early Access Framework

Panellists weighted the quantitative criteria included in the pilot framework during the second session. According to the results of the weighting, none of the criteria was considered futile, being the lowest relative importance for plausibility, with 3.6 points out of 5.

**Figure 1:**
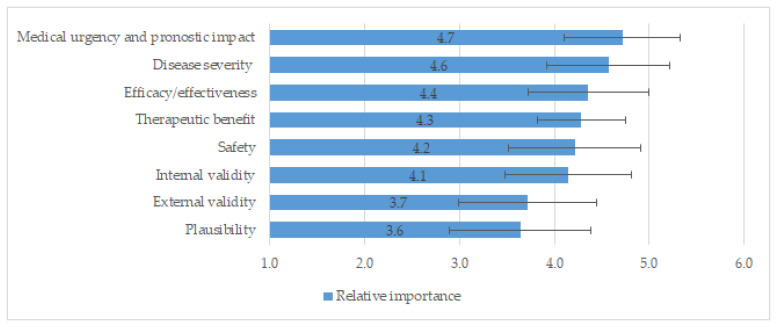
Mean (SD) of relative importance rated by participants to final early access framework.

**Figure 2:**
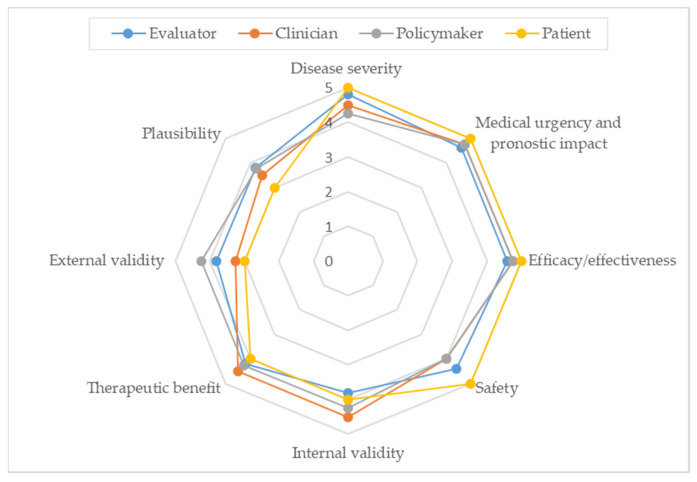
Relative importance according to professional profile.

Since the framework underwent relevant changes during the reflective discussion and deliberation of the burosumab case study, it was required to rate the framework once again. Figure 1 shows the relative importance of each criterion included in the final framework. The most important criteria (mean ± SD) were “urgency” (4.7 ± 0.6), followed by “severity of disease” (4.6 ± 0.6), and “efficacy” (4.4 ± 0.6). The least important criteria were “external validity” (3.7 ± 0.7) and “plausibility” (3.6 ± 0.7). The SD were low (≤0.7) in all criteria presuming a high agreement among panellists. Figure 2 shows the relative importance of each criterion according to the professional profile of the participants.

### 3.3. Value Contribution of a Case Study

**Figure 3:**
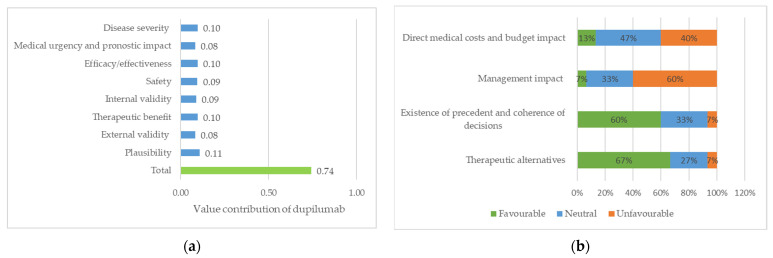
Dupilumab for the treatment of a paediatric patient with severe atopic dermatitis: (**a**) value of contribution of quantitative criteria; (**b**) impact of qualitative criteria (percentage of members who assigned a favourable, neutral and unfavourable).

In the third session, 15 of the 18 participants agreed to the final framework by scoring the dupilumab case study for a paediatric patient with severe atopic dermatitis. As shown in Figure 3a, the most valued quantitative criterion was “plausibility”, followed by “severity of disease”, “efficacy” and “therapeutic benefit”; the less valued criteria were “external validity” and “urgency”. The overall assessed value contribution of dupilumab for the paediatric patient was 0.74 (maximum value of 1). Regarding the contextual tool (Figure 3b), “lack of therapeutic alternatives for the patient” and “the existence of precedents” showed a positive impact, while “management impact” suggested a negative impact in supporting the approval.

### 3.4. Re-Test of the Early Access Framework

After using the final framework for six months by the CAMSE in routine setting, a re-test was done to check consistency in application with the participation of all the members, using volanesorsen for the treatment of FCS as a case study. The patient showed a high level of triglycerides even with good adherence to hypolipemiant treatments and strict diet and had had several acute pancreatitis episodes that had led to exocrine and endocrine pancreatic insufficiency.

For the first reflective MCDA deliberation, CAMSE members expressed concern regarding the safety profile and efficacy uncertainties of volanesorsen, being the final value contribution 0.67 points. The contextual tool, “lack of therapeutic alternatives” swung towards a positive impact for the approval (92%), while “direct medical costs” had an unfavourable impact (100%). Finally, CAMSE gave a favourable recommendation for use.

Six months later, the CAMSE members re-assessed and scored again the evidence matrix of volanesorsen. The value contribution of the re-test was 0.69 points, with no significant differences in the application of the criteria or the reflective discussion.

## 4. Discussion

In the present study, we describe that the MCDA methodology approach is a feasible tool applicable to the assessment of patients’ individual requests for early access medicines. Importantly, the re-test of the MCDA framework developed suggests that the method is consistent and replicable on time at the CAMSE level.

MCDA has been used in a diverse range of health care decisions and levels [18,32]. At the macro level, it has been mostly used by HTA bodies to rank and set priorities among therapeutic alternatives and inform reimbursement decisions [20,21,22,23,25,33], and it has also has been proposed for the benefit–risk assessment to support marketing authorisation [34]. At lower decision levels, MCDA has been used to inform hospital decision making to incorporate medicines into the hospital formulary [24] and also to support shared decision making involving the patient’s voice [35]. To our knowledge, this is the first study to use the MCDA tool to support decision making for individual requests that are assessed by a Committee. This fact prompted the need to adapt each criterion to the particularities and requirements of the situation. Several sessions with the CAMSE revealed that multiple factors might affect a decision of exceptional access to a medicine/indication that is still not available in Spain. A set of criteria to assess a request were consensually agreed and consisted of eight quantitative criteria (“severity of disease”, “urgency”, “efficacy”, “safety”, “internal validity”, “plausibility”, “external validity” and “therapeutic benefit”) and four contextual criteria (“therapeutic alternatives”, “existence of precedent and coherence”, “management impact” and “direct medical costs and budget impact”).

Some of the criteria directly reflect established prerequisites of the Spanish legislation for early access to treatment. That was the case for the “therapeutic alternative” criterion since the law limits MSS to situations where no available alternatives are commercialised or available. The criterion was rapidly accepted as relevant; however, its definition was widely discussed among participants. A central issue at the debate was whether off-label use of medicines should be considered as an alternative therapy, showing differences between regulatory and clinical perspectives. After deliberation, participants agreed that off-label drugs could be an alternative therapy only when the proposed medicine was commercially available for an authorised indication, and its use in the studied indication was well established in clinical practice and supported by scientific evidence. On the other hand, CAMSE members expressed their discomfort in scoring the criterion on a scale from 0 to 5 during the test of the pilot framework in the first case study, and therefore, it was transferred to the contextual tool.

Because of the nature of the MSS requests, with no available comparable alternatives, efficacy and safety were not evaluated as comparative criteria but included only positive scores scales. This fact contrasts with the EVIDEM framework, whose aim usually consist of prioritising treatments for the same indication.

As stated in Marsh et al., several properties have to be taken into consideration while selecting the criteria for the MCDA framework: completeness, non-redundancy and non-overlap and preference independence [30].

Capturing all factors relevant to the decision met the completeness requirement. The requirement of non-redundancy was also met, as no criteria were removed or considered unimportant, with all criteria being weighted with more than 3.5 points out of 5. Preference independence was also achieved. It was debated whether “urgency” and “severity” could somehow overlap, but it was agreed that while “urgency” clearly defines a therapeutic window for the use of a medicine, “severity” establishes the functional impact of the disease on the patient. An example might be a request for advanced therapy for the treatment of congenital retinal dystrophy that can only be used while the patient has enough functioning cells in the retina. The request would be valued as urgent because not giving the therapy now would result in irreversible damage and thus would have a negative prognostic impact for the patient, but the situation of the patient may not be still severe because the disease has not yet been caused major vision loss.

As expected, “urgency” and “severity of disease” at the time of request were the most important criteria according to the weighting. Both criteria show the reasoning that the request must justify the necessity of the treatment through a non-standard route under the ethical foundation for alleviating suffering in those who are worst-off and the cost of opportunity that lags behind the delay of treatment when it may cause a negative impact on the person in need, and is based on the non-maleficence ethical principle. We encountered “efficacy” and “therapeutic benefit” also ranked between the highest weights, revealing the beneficence principle of deontology. These criteria were followed by safety, also as a non-maleficence principle. Criteria related to the quality of the study and extrapolation of results into the individual patient, as well as evaluating the likelihood of the product to show beneficial effects in the patient, were the least important criteria.

Weighting involves eliciting stakeholders’ preferences between criteria. The variations in weights in the quantitative criteria observed in the study according to participants’ profile reveals differences in their personal values [20] and highlights the critical impact of a committee composition. Although the variability was low, showing considerable agreement between participants, some differences are noticeable. For instance, amongst the eight quantitative criteria, the greatest weighting for “severity”, “urgency”, “efficacy” and “safety” was given by the patient representative, while “internal validity” and “therapeutic benefit” had the highest rating by clinicians and “external validity” and “plausibility” by policymakers. To note, the patient representative considered “external validity” and “plausibility” less important than both policymakers and evaluators.

The inclusion of a variety of profiles is necessary in order to identify and capture the overall range of health perception values [19,20]. CAMSE is a multidisciplinary advisory Committee; nonetheless, two additional stakeholders were invited to participate in the panel, to account for the fact that some degree of decision making is done before MSS requests arrive in the Committee so that a better-balanced composition with regard to the entire process was provided.

Regarding the contextual tool, the EVIDEM framework proposes a positive, negative and neutral rating, but the meaning of the wording was not clear to the participants and therefore changed to a more explicit concept (i.e., favourable, unfavourable or neutral impact). Differences in the rating on the contextual tool help identify those aspects of each decision that may profit from further discussion.

The overall value contribution obtained for the hypothetical request of dupilumab for the treatment of a paediatric patient was 0.74, which could reflect a high added value in the particular case study. However, because the core model has been adapted so that criteria included definitions and ratings differ between studies, value estimates cannot be compared with other previously obtained using EVIDEM methodology [27].

Previous studies in CatSalut have focused on the use of MCDA for the evaluation of orphan drugs in the HTA regional context [27,29]. Even though early access may often be requested in cases of high unmet medical need, such as rare diseases, other areas such as oncology and several immune-mediated diseases take special relevance in MSS. This is partly due to a high speed of development arising from advances in molecular biotechnology and the development of highly targeted medicinal products [8,36]. In this context, CAMSE assesses very heterogeneous applications, so the use of an already existing MCDA framework specific for orphan drugs would not be appropriate. The two medicines selected to work on the framework were intendedly an orphan medicinal product and a non-orphan treatment to reflect the scope of CAMSE and to verify that the early access framework provided common criteria, relevant regardless of the type of drug.

A distinctive feature of our study is that we applied the MCDA early-access framework to support treatment decisions for a particular patient in exceptional situations where a clinician believes that the clinical situation requires a medicine prescribed outside the standard mechanisms. In such cases, and due to the potential clinical and economic risks of their use, as well as because precedents are generated, every single case is considered carefully before a recommendation is issued. Thus, in contrast to the majority of the studies describing the use of MCDA for decisions addressed to wider populations, our goal for MCDA application in early access to NAM-PR was to understand the value of a treatment for an individual and the future impact of the decision in the system, being both a patient and medicine-focused assessment. The MCDA approach described in this study is intended to improve traceability of decisions and gain consistency, thus avoiding arbitrariness or contradictions in time or across diseases; however, because the focus are single treatments in exceptional circumstances, it is not intended to substitute meetings nor to avoid the need for case-by-case assessment by the CAMSE.

The current study has some limitations. Although the panel consisted of 18 participants and intended to include diverse perspectives, only one patient and one representative of bioethical perspective participated in the study, potentially underrepresenting these views as opposed to other profiles. Patient representatives are present in all Catalan medicines committees, their role being that of providing user’s perspective and transversal patient advocacy for all diseases, rather than providing detailed information on the disease discussed. The patient in the panel has been trained on methodology and regulation, is familiar with the language and terms used in drug appraisals, and together with the bioethicist trained person, both actively contribute with perspectives on subjective suffering and opportunity costs, equity, justice and need of transparency and accountability of the committee. Another limitation is that the number of experts included was not powered to measure variations across profiles. In addition, even though the early access pilot framework was developed to give answers to different types of medicines and situations, we used only two medicines and hypothetical individual requests in the exercise and a third one in the re-test.

Recommendations for the use of MCDA as a tool to support decisions in product development focus in early HTA, with few experiences reported [37]. There is a lack of methodological references to methods aimed to inform decision making on early access to medicines for those patients in need in European countries. We have developed an MCDA framework that has now been applied by the committee for some months since inception, and we are gathering experience and data whose analysis will be of interest in the upcoming future. To further develop the framework and encourage and consolidate its use, a wider evidence matrix from early access drugs has to be tested within the CAMSE, as well as other special situations such as off-label use of innovative medicines. Hence, the early-access framework developed in the Catalan context could serve as a starting methodological approach for other countries facing similar challenges.

## 5. Conclusions

In Spain, access to non-authorised and pre-reimbursed medicines/indications represents an exceptional situation of special complexity since their use and financing is not clearly defined. Lack of methodological reference causes health policymakers to deal with many uncertainties. The present study suggests that reflective MCDA may support systematic decision making by facilitating discussion for consensus on recommendations for the early access of heterogeneous, complex and sensitive MSS requests in CatSalut. The use of a structured methodology as a decision-making support tool may improve consistency, robustness and transparency of decisions on the priority of treatments for patients in need while keeping compliance with the regulatory and financing aspects and foreseeing potential impact on future commercial access. By improving predictability, MCDA may allow better communication of the rationale behind decisions and could be useful to manage patients’ and clinicians’ expectations.

## Figures and Tables

**Table 1:** Criteria considered in the pilot early access framework.

Possible Criteria	Type of Criteria	Source
Disease severity at the time of application	Quantitative	EVIDEM framework v.4 (10th Edition)
Medical urgency to receive treatment and expected prognostic impact at the time of application	Quantitative	Early access specific criteria
Therapeutic alternatives for the patient	Quantitative	Early access specific criteria
Efficacy/effectiveness	Quantitative	EVIDEM framework v.4 (10th Edition); adapted non-comparative
Safety	Quantitative	EVIDEM framework v.4 (10th Edition); adapted non-comparative
Quality of evidence	Quantitative	EVIDEM framework v.4 (10th Edition)
Therapeutic benefit	Quantitative	EVIDEM framework v.4 (10th Edition)
Plausibility	Quantitative	Early access specific criteria
Existence of precedent and coherence of decisions	Qualitative	Early access specific criteria
Management impact	Qualitative	Early access specific criteria
Direct medical costs	Qualitative	EVIDEM framework v.4 (10th Edition)

**Table 2:** Final MCDA core model and MCDA contextual tool for early access framework.

Criteria	Subcriteria	Scoring Scale
Core Model		
Disease severity at the time of application	Effect of the disease on patient’s morbidity at the time of application Functional impact on patient	0: No functional affectation → 5: Permanent disability or critical situation or vital support requirement
Medical urgency to receive a treatment and expected prognostic impact	Speed progression Opportunity period for treating the patient Prognostic impact of not receiving the treatment	0: No urgent, slow progression or no sequels → 5: Very urgent, fulminant progression or severe and permanent sequels
Efficacy/effectiveness	End points used Magnitude of health gain Duration of health gain	0: Not effective or no data → 5: Very effective
Safety	Frequent adverse events Serious adverse events Fatal adverse events Discontinuation due to adverse events	0: Frequent serious adverse effects, fatal adverse effects or no data on safety → 5: Very safe, mild adverse effects
Internal validity	Quality of evidence Robustness Completeness of reporting Type of evidence	0: Low internal validity → 5: High internal validity
Therapeutic benefit	Type of therapeutic benefit expected of the intervention	0: No therapeutic benefit → 5: Cure
External validity	Representativeness and relevance of the results of the studies with respect to the case requested	0: Low external validity → 5: High external validity
Plausibility	Association between the mechanism of action of the drug and the expected effect on the patient	0: Not plausible or 5: Plausible
Contextual Tool		
Therapeutic alternatives	Available therapeutic alternatives to treat the patient	Favourable, neutral, unfavourable
Existence of precedent and coherence of decisions	Decision on previous applications received for the same drug/indication Number of treatments initiated in Spain	Favourable, neutral, unfavourable
Influence of the decision at a policy level (management impact)	Possible influence of the decision taken on the negotiation of the price and financing of the drug.	Favourable, neutral, unfavourable
Direct medical costs and budget impact	Treatment cost/patient Other medical costs for treating the patient Potential budget impact	Favourable, neutral, unfavourable

## Data Availability

Not applicable.

## Supplementary Materials

The following are available online at https://www.mdpi.com/article/10.3390/jcm11051353/s1, Table S1: Definitions of the criteria in the early access framework.

## References

[1] (2001). Directive 2001/83/EC of the European Parliament and of the Council of 6 November 2001 on the Community Code Relating to Medicinal Products for Human Use.

[2] Henshall C., Mardhani-Bayne L., Frønsdal K.B., Klemp M. (2011). Interactions between Health Technology Assessment, Coverage, and Regulatory Processes: Emerging Issues, Goals, and Opportunities. Int. J. Technol. Assess. Health Care.

[3] Patil S. (2016). Early Access Programs: Benefits, Challenges, and Key Considerations for Successful Implementation. Perspect. Clin. Res..

[4] Angelis A., Lange A., Kanavos P. (2018). Using Health Technology Assessment to Assess the Value of New Medicines: Results of a Systematic Review and Expert Consultation across Eight European Countries. Eur. J. Health Econ..

[5] World Health Organization—WHO (2018). Medicines Reimbursement Policies in Europe.

[6] European Medicines Agency (EMA). https://www.ema.europa.eu/en/human-regulatory/marketing-authorisation/conditional-marketing-authorisation.

[7] Godman B., Bucsics A., Bonanno P.V., Oortwijn W., Rothe C.C., Ferrario A., Bosselli S., Hill A., Martin A.P., Simoens S. (2018). Barriers for Access to New Medicines: Searching for the Balance between Rising Costs and Limited Budgets. Front. Public Health.

[8] Wilking N., Bucsics A., Kandolf Sekulovic L., Kobelt G., Laslop A., Makaroff L., Roediger A., Zielinski C. (2019). Achieving Equal and Timely Access to Innovative Anticancer Drugs in the European Union (EU): Summary of a Multidisciplinary CECOG-Driven Roundtable Discussion with a Focus on Eastern and South-Eastern EU Countries. ESMO Open.

[9] Scavone C., di Mauro G., Mascolo A., Berrino L., Rossi F., Capuano A. (2019). The New Paradigms in Clinical Research: From Early Access Programs to the Novel Therapeutic Approaches for Unmet Medical Needs. Front. Pharmacol..

[10] Krendyukov A. (2020). Early Access Provision for Innovative Medicinal Products in Oncology: Challenges and Opportunities. Front. Oncol..

[11] Whitfield K., Huemer K.H., Winter D., Thirstrup S., Libersa C., Barraud B., Kubiak C., Stankovski L., Grählert X., Dreier G. (2010). Compassionate Use of Interventions: Results of a European Clinical Research Infrastructures Network (ECRIN) Survey of Ten European Countries. Trials.

[12] Löblová O., Csanádi M., Ozierański P., Kaló Z., King L., McKee M. (2019). Alternative Access Schemes for Pharmaceuticals in Europe: Towards an Emerging Typology. Health Policy.

[13] Ministerio de Sanidad y Política Social (2009). Real Decreto 1015/2009, de 19 de Junio, Por El Que Se Regula La Disponibilidad de Medicamentos En Situaciones Especiales. Bol. Estado.

[14] OECD Health Division (2020). Addressing Challenges in Access to Oncology Medicines Analytical Report. https://www.oecd.org/health/health-systems/Addressing-Challenges-in-Access-to-Oncology-Medicines-Analytical-Report.pdf.

[15] Consulta Pública Previa Proyecto de Real Decreto Por el Que se Regula la Disponibilidad de Medicamentos en Situaciones Especiales. https://www.mscbs.gob.es/en/normativa/docs/RD_REGULACION_DISPONIBILIDAD_MEDICAMENTOS_SITUACIONES_ESPECIALES.pdf.

[16] Danés I., Agustí A., Vallano A., Alerany C., Martínez J., Bosch J.A., Ferrer A., Gratacós L., Pérez A., Olmo M. (2014). Outcomes of Off-Label Drug Uses in Hospitals: A Multicentric Prospective Study. Eur. J. Clin. Pharmacol..

[17] CatSalut (2018). Resolution Creating the Advisory Council on Medicines in Special Situations (CAMSE). https://catsalut.gencat.cat/web/.content/minisite/catsalut/proveidors_professionals/normatives_instruccions/2019/resolucio-camse.pdf.

[18] Thokala P., Devlin N., Marsh K., Baltussen R., Boysen M., Kalo Z., Longrenn T., Mussen F., Peacock S., Watkins J. (2016). Multiple Criteria Decision Analysis for Health Care Decision Making—An Introduction: Report 1 of the ISPOR MCDA Emerging Good Practices Task Force. Value Health.

[19] Baltussen R., Paul Maria Jansen M., Bijlmakers L., Grutters J., Kluytmans A., Reuzel R.P., Tummers M., van der Wilt G.J. (2017). Value Assessment Frameworks for HTA Agencies: The Organization of Evidence-Informed Deliberative Processes. Value Health.

[20] Goetghebeur M.M., Wagner M., Khoury H., Levitt R.J., Erickson L.J., Rindress D. (2012). Bridging Health Technology Assessment (HTA) and Efficient Health Care Decision Making with Multicriteria Decision Analysis (MCDA): Applying the Evidem Framework to Medicines Appraisal. Med. Decis. Mak..

[21] Elvira D., Obach M., Pontes C. (2021). Description of the Use of Multicriteria to Support Pricing and Reimbursement Decisions by European Health Technology Assessment Bodies. BMC Health Serv. Res..

[22] Radaelli G., Lettieri E., Masella C., Merlino L., Strada A., Tringali M. (2014). Implementation of Eunethta Core Model^®^ in Lombardia: The VTS Framework. Int. J. Technol. Assess. Health Care.

[23] Howard S., Scott I.A., Ju H., McQueen L., Scuffham P.A. (2019). Multicriteria Decision Analysis (MCDA) for Health Technology Assessment: The Queensland Health Experience. Aust. Health Rev..

[24] Roldán Ú.B., Badia X., Marcos-Rodríguez J.A., De La Cruz-Merino L., Gómez-González J., Melcón-De Dios A., De La Caraballo-Camacho M.O., Cordero-Ramos J., Alvarado-Fernández M.D., Galiana-Auchel J.M. (2018). Multi-Criteria Decision Analysis as a Decision-Support Tool for Drug Evaluation: A Pilot Study in a Pharmacy and Therapeutics Committee Setting. Int. J. Technol. Assess. Health Care.

[25] de Andrés-Nogales F., Cruz E., Calleja M.Á., Delgado O., Gorgas M.Q., Espín J., Mestre-Ferrándiz J., Palau F., Ancochea A., Arce R. (2021). A Multi-Stakeholder Multicriteria Decision Analysis for the Reimbursement of Orphan Drugs (FinMHU-MCDA Study). Orphanet J. Rare Dis..

[26] Adunlin G., Diaby V., Montero A.J., Xiao H. (2015). Multicriteria Decision Analysis in Oncology. Health Expect..

[27] Guarga L., Badia X., Obach M., Fontanet M., Prat A., Vallano A., Torrent J., Pontes C. (2019). Implementing Reflective Multicriteria Decision Analysis (MCDA) to Assess Orphan Drugs Value in the Catalan Health Service (CatSalut). Orphanet J. Rare Dis..

[28] Goetghebeur M.M., Wagner M., Khoury H., Levitt R.J., Erickson L.J., Rindress D. (2008). Evidence and Value: Impact on DEcisionMaking-The EVIDEM Framework and Potential Applications. BMC Health Serv. Res..

[29] Gilabert-Perramon A., Torrent-Farnell J., Catalan A., Prat A., Fontanet M., Puig-Peiró R., Merino-Montero S., Khoury H., Goetghebeur M.M., Badia X. (2017). Drug Evaluation and Decision Making in Catalonia: Development and Validation of a Methodological Framework Based on Multi-Criteria Decision Analysis (MCDA) for Orphan Drugs. Int. J. Technol. Assess. Health Care.

[30] Marsh K., Ijzerman M., Thokala P., Baltussen R., Boysen M., Kaló Z., Lönngren T., Mussen F., Peacock S., Watkins J. (2016). Multiple Criteria Decision Analysis for Health Care Decision Making-Emerging Good Practices: Report 2 of the ISPOR MCDA Emerging Good Practices Task Force. Value Health.

[31] BIFIMED: Search Engine for Information on the Funding Status of Medicines-Health Ministry. Madrid. https://www.mscbs.gob.es/profesionales/medicamentos.do?metodo=buscarMedicamentos.

[32] Jakab I., Németh B., Elezbawy B., Karadayı M.A., Tozan H., Aydın S., Shen J., Kaló Z. (2020). Potential Criteria for Frameworks to Support the Evaluation of Innovative Medicines in Upper Middle-Income Countries—A Systematic Literature Review on Value Frameworks and Multi-Criteria Decision Analyses. Front. Pharmacol..

[33] Tony M., Wagner M., Khoury H., Rindress D., Papastavros T., Oh P., Goetghebeur M.M. (2011). Bridging Health Technology Assessment (HTA) with Multicriteria Decision Analyses (MCDA): Field Testing of the EVIDEM Framework for Coverage Decisions by a Public Payer in Canada. BMC Health Serv. Res..

[34] Angelis A., Phillips L.D. (2021). Advancing Structured Decision-Making in Drug Regulation at the FDA and EMA. Br. J. Clin. Pharmacol..

[35] Badia X., Aguarón A., Fernández A., Gimón A., Nafria B., Gaspar B., Guarga L., Gálvez M., Fuentes M., Paco N. (2019). Patient Involvement in Reflective Multicriteria Decision Analysis to Assist Decision Making in Oncology. Int. J. Technol. Assess. Health Care.

[36] McInnes I.B., Gravallese E.M. (2021). Immune-Mediated Inflammatory Disease Therapeutics: Past, Present and Future. Nat. Rev. Immunol..

[37] Angelis A., Thursz M., Ratziu V., O’Brien A., Serfaty L., Canbay A., Schiefke I., Costa J.B., Lecomte P., Kanavos P. (2020). Early Health Technology Assessment during Nonalcoholic Steatohepatitis Drug Development: A Two-Round, Cross-Country, Multicriteria Decision Analysis. Med. Decis. Mak..

