# Supplementary material for: Early Access to Medicines: Use of Multicriteria Decision Analysis (MCDA) as a Decision Tool in Catalonia (Spain)
Source: J Clin Med. 2022 Mar 1;11(5):1353. doi: 10.3390/jcm11051353 (PMC8910942; doi:10.3390/jcm11051353)
Supplement: Supplementary file 1 [file jcm-11-01353-s001.zip › jcm-1585349-supplementary.pdf]

Suplemmentary material

Table S1. Criteria and definitions of the early access framework

Criteria	Definition
Disease severity at the time of application	Severity of the patient's condition at the time when the medicine is requested in special situations. This includes the patient's situation from greater to lesser impact (e.g.: need for life support, serious organic dysfunction, major functional impairment, inability to carry out daily activities independently, among others).
Medical urgency to receive a treatment and expected prognostic impact	The assessment of the speed of progression of the disease and/or associated complications at the time of the request and the time window in which treating the patient could have a clinical benefit (e.g.: immediate treatment is required because the patient has a fulminant progression or otherwise treatment can wait because the progression of the disease is slow). Impact of delaying the start of treatmentin the prognosis of the patient and the consequences that this may cause.
Efficacy/effectiveness	Capacity of the requested medicine to generate a clinically relevant change in the study endpoints (same pathology of the requested case). Includes data on efficacy and/or effectiveness and duration of efficacy. Considers also data reported by patients (e.g. quality of life, etc.).
Safety	Short- and long-term safety results of the medicine requested. Consider the frequency, severity and reversibility of the adverse effects as well as the uncertainties related to the available evidence for the same pathology of the requested case.
Internal validity	Quality of the studies, in which data are relevant (in terms of type of patients, stage of disease, comparator treatments, main variable, results, etc.) and valid in terms of scientific standards (i.e. study design, etc.) and robust (concordance of results between studies). If necessary, include types of evidence and consideration of uncertainty(ies) (e.g. contradictory results between studies, limited number of studies and patients).
Therapeutic benefit	Assessment of the type of clinical benefit provided by the medicine (e.g. alleviation of symptoms, prolongation of life, cure, adverse events avoided [exacerbations], etc.) and to what extent it resolves the patient's clinical situation.
External validity	Applicability of the results obtained from the clinical studies in the assessment of the requested case. Consider whether the patient for whom the application is made is representative of those included in the studies in terms of baseline and pathogenic characteristics (e.g. age, sex, diagnosis, concomitant diseases, stage of the disease, mutations, previous treatments used, among others).
Plausibility	Based on the request assessed and considering the available scientific evidence, state whether the treatment is likely to be beneficial in the subpopulation represented by the patient for whom the treatment is requested.
Therapeutic alternatives	Availability of alternative therapies to improve, alleviate or cure the disease or absence of alternative treatments in the requested indication. It also includes an exhaustion of therapeutic alternatives due to its lack of effectiveness, possible toxicity, non-tolerance or contraindication.

	Consider therapeutic alternative to those treatments that have the same type of benefit (curative, palliative or preventive). Off-label use of medicines will be considered therapeutic alternatives when their use is well established in clinical practice (based on robust scientific evidence) and included in clinical practice guidelines and the product is marketed and reimbursed in Spain.<br>Treatments with an explicit negative price and reimbursement decision by the Ministry of Health will not be considered as alternatives.<br>Include whether the patient has been considered for inclusion in a clinical trial.
Existence of precedent and coherence of decisions	Assessment of the requests previously received and the representativeness of the case with respect to the new case. Take into account the context in which previous decisions were taken. Assess the change in the regulatory framework and the evidence available with respect to the previous decision.
Influence of the decision at a policy level (Management impact)	Influence of the decision taken on the initiation of treatment on a possible negotiation of price and reimbursement between the Ministry of Health and the laboratory responsible for marketing the drug on the basis of:<br>- clinical representativeness of the case with respect to the evaluated indication<br>- origin of a clinical trial<br>- potential number of patients for whom this case may represent a precedent
Direct medical costs and budget impact	Cost of the treatment requested for the patient during the period of time corresponding to the indication (acute or chronic) and other medical costs associated with the management of the patient as a consequence of receiving the treatment (e.g. administration cost in hospital setting).<br>Consider the costs of alternative therapies if necessary.<br>Budget impact that the beginning of the treatment may represent depending on the number of patients that could be susceptible to receive it.
